# Platinum(II)-Acyclovir Complexes: Synthesis, Antiviral and Antitumour Activity

**DOI:** 10.1155/MBD.1995.249

**Published:** 1995

**Authors:** M. Coluccia, A. Boccarelli, C. Cermelli, M. Portolani, G. Natile

**Affiliations:** 1 Dipartimento di Scienze Biomediche e Oncologia Umana Piazza G. Cesare11 Bari 1-70124 Italy; 2 Dipartimento di Scienze Biomediche Università di Modena Modena Italy; 3 Dipartimento Farmaco-Chimico Università di Bari Bari Italy

## Abstract

A platinum(II) complex with the antiviral drug acyclovir was synthesized and its antiviral and anticancer properties were investigated in comparison to those of acyclovir and cisplatin. The platinum-acyclovir complex maintained the antiviral activity of the parent drug acyclovir, though showing a minor efficacy on a molar basis (ID_50_  =   7.85 and 1.02 μΜ for platinum-acyclovir and cisplatin, respectively). As anticancer agent, the platinum-acyclovir complex was markedly less potent than cisplatin on a mole-equivalent basis, but it was as effective as cisplatin when equitoxic dosages were administered *in vivo* to P388 leukaemia-bearing mice (%T/C = 209 and 211 for platinum-acyclovir and cisplatin, respectively). The platinum-acyclovir complex was also active against a cisplatin-resistant subline of the P388 leukaemia (%T/C = 140), thus suggesting a different mechanism of action. The DNA interaction properties (sequence specificity and interstrand cross-linking ability) of platinum-acyclovir were also investigated in comparison to those of cisplatin and [Pt(dien)Cl]^+^, an antitumour-inactive platinum-triamine compound. The results of this study point to a potential new drug endowed, at the same time, with antiviral and anticancer activity and characterized by DNA interaction properties different from those of cisplatin.


### PLATINUM(II)-ACYCLOVIR COMPLEXES: SYNTHESIS, ANTIVIRAL AND ANTITUMOUR ACTIVITY

M. Coluccia[1], A. Boccarelli[1], C. Cermelli[2], M. Portolani[2], and G. Natile[3]

[1] Dipartimento di Scienze Biomediche e Oncologia Umana, Piazza G. Cesare 11, I-70124 Bari, Italy
[2] Dipartimento di Scienze Biomediche, Università di Modena, Italy
[3] Dipartimento Farmaco-Chimico, Università di Bari, Italy

### ABSTRACT

A platinum(II) complex with the antiviral drug acyclovir was synthesized and its antiviral and anticancer properties were investigated in comparison to those of acyclovir and cisplatin. The platinum-acyclovir complex maintained the antiviral activity of the parent drug acyclovir, though showing a minor efficacy on a molar basis ($ID_{50}$ = 7.85 and 1.02 µM for platinum-acyclovir and cisplatin, respectively). As anticancer agent, the platinum-acyclovir complex was markedly less potent than cisplatin on a mole-equivalent basis, but it was as effective as cisplatin when equitoxic dosages were administered *in vivo* to P388 leukaemia-bearing mice (%T/C = 209 and 211 for platinum-acyclovir and cisplatin, respectively). The platinum-acyclovir complex was also active against a cisplatin-resistant subline of the P388 leukaemia (%T/C = 140), thus suggesting a different mechanism of action. The DNA interaction properties (sequence specificity and interstrand cross-linking ability) of platinum-acyclovir were also investigated in comparison to those of cisplatin and $[Pt(dien)Cl]^+$, an antitumour-inactive platinum-triamine compound. The results of this study point to a potential new drug endowed, at the same time, with antiviral and anticancer activity and characterized by DNA interaction properties different from those of cisplatin.

### INTRODUCTION

Acyclovir (9-(2-hydroxyethoxymethyl)guanine, acycloguanosine) and cisplatin (*cis*-diaminedichloroplatinum(II), *cis*-DDP) are two drugs currently used in antiviral and anticancer therapy, respectively.

Acyclovir is a nucleoside analogue with potent activity towards herpes virus infections. Acyclovirtriphosphate is preferentially formed in infected cells and inhibits viral DNA synthesis either selectively interfering with viral DNA polymerase or leading to premature chain termination (1).

The metal-coordinating properties of acyclovir are of current interest (2-5) from a mechanistic point of view, some DNA polymerases containing ($Zn^{2+}$) and/or being activated by metal ions ($Mg^{2+}$, $Mn^{2+}$ or $Co^{2+}$). Moreover, metal complexes of acyclovir may exhibit antiviral activity different from that of the free ligand (6).

Cisplatin is one of the most successful antitumour drugs developed in recent years and numerous studies have revealed that its cytotoxic activity depends upon the interaction with cellular DNA. Cisplatin and related platinum(II) analogues coordinate two neighbouring purines, thus producing bifunctional lesions able to inhibit DNA replication and/or transcription (7). Platinum(II) complexes with nucleosides and nucleoside analogues are also of current interest as model compounds; moreover, platinum(II) complexes with nucleotide derivatives of formula $[PtCl(NH_3)_2(Am)]^+$ (Am = pyrimidine or purine derivative) have recently demonstrated activity in preclinical tumour screens, thus suggesting that also monofunctional DNA lesions might determine a cytotoxic effect (8).

In our search on the platinum-coordination properties of acyclovir we synthesized a compound derived from cisplatin. The antiviral and antitumour properties of such a compound, *cis*-$[PtCl(NH_3)_2(acyclovir)]NO_3$ have been investigated and compared to those of acyclovir and cisplatin. Moreover, preliminary studies on the DNA interaction properties of this platinum-acyclovir complex are reported. The results point to a potential new drug endowed, at the same time, with antiviral and antitumour properties.

### MATERIALS AND METHODS

*Synthesis of the complexes.* 9-(2-hydroxyethoxymethyl)guanine (acyclovir, $L^1$), 9-(2-acethoxyethoxymethyl)guanine (monoacetylacyclovir, $L^2$) and 9-(2-acetoxyethoxymethyl)-N(2)-acetylguanine (diacetylacyclovir, $L^3$) were prepared by the method of Matsumoto et al. (9).

Preparation of *cis*-$[PtCl_2(NH_3)(L^1)]$ (**1**) . The compound was prepared by the method of Rochon and Kong (10) by bridge splitting with acyclovir of the dimer $[PtI_2(NH_3)]_2$ obtained from reaction of *cis*-$[PtI_2(NH_3)_2]$ with perchloric acid. The chloro compound could be obtained from the corresponding iodo species by precipitating the iodo ligands with a silver salt and adding KCl. The desidered product was formed in less than 50% yield and was accompanied by two major byproducts; the low solubility prevented their separation and full characterization. Anal. Calcd for $C_8H_{14}N_6O_3PtCl_2$: C, 12.8; H, 1.9; N, 11.3. Found: C, 13.2; H, 2.0; N, 11.4%.

Preparation of *cis*-$[PtCl_2L_2]$ (L = $L^2$, **2**; $L^3$, **3**). The complexes were prepared by reaction of $K_2[PtCl_4]$ and L in 1:2 molar ratio. Thus $K_2[PtCl_4]$ (0.20 g, 0.5 mmol) in water (50 ml) was treated with L (1 mmol) and the suspension was left to stir at room temperature for 3 d. The yellow precipitate was collected, washed with water, then with methanol, and finally with ether. Yield 80%. Found: C, 30.6; H, 3.3; Cl, 9.0; N, 17.6. Calc. for $C_{20}H_{26}Cl_2N_{10}O_8Pt$, **2**: C, 30.0; H, 3.3; Cl, 8.9; N, 17.5. Found: C, 32.3; H, 3.3; Cl, 8.3; N, 16.0. Calc. for $C_{24}H_{30}Cl_2N_{10}O_{10}Pt$, **3**: C, 32.6; H, 3.4; Cl, 8.0; N, 15.8%.

Preparation of *cis*-$[PtCl(NH_3)_2(L^1)]NO_3$ (**4**). This complex was prepared from *cis*-$[PtCl_2(NH_3)_2]$ and $L^1$ according to the method of Hollis *et al.* (11). *Cis*-$[PtCl_2(NH_3)_2]$ (0.30 g, 1 mmol) and $AgNO_3$ (0.15 g, 0.9 mmol) were stirred in dimethylformamide (DMFA) (30 ml) for 1 d. The resulting mixture was filtered and the stoichiometric amount of $L^1$ (0.22 g, 1 mmol) was added to the filtrate. Stirring was resumed and continued for 1 d. After a second filtration, the solution was taken to dryness and the residue crystallized from water. Yield 70%. Found: C, 17.4; H, 3.1; Cl, 6.7; N, 20.2; Calc. for $C_8H_{17}ClN_8O_6Pt$, **4**: C, 17.4; H, 3.1; Cl, 6.4; N, 20.3%.

*Physical Measurements.* Infrared spectra in the range 4000-400 $cm^{-1}$ were recorded as KBr pellets; spectra in the range 400-200 $cm^{-1}$ were recorded as Polythene pellets on Perkin-Elmer 283 and FT 1600 spectrophotometers. Proton NMR spectra were obtained with Varian XL200 and Bruker AM 300 spectrometers.

*Antiviral activity.* The antiviral activity was evaluated *in vitro* by the plaque reduction assay. For this purpose, 24 h growth VERO cell monolayers in 60 mm Petri dishes were infected with 0.5 ml of a Herpes Simplex-1 virus stock solution containing 500 Plaque Forming Units/ml. After 1 h incubation at 37 °C the dishes were washed twice with sterile

PBS and a semisolid maintenance medium containing the drug under study was added. Each drug concentration was tested in triplicate and in three control cultures the cell medium contained only the appropriate amount of drug solvent (DMSO). After 48 h incubation, cell monolayers were fixed with methanol for 20 min, stained with Giemsa stain for 15 min and then the cytolysis plaques were counted. The 50% inhibitory concentrations ($ID_{50}$) were derived by interpolation from a log-linear plot of concentration-per cent plaque reduction outcomes.

*Antitumour activity.* The P388 murine leukaemia was used to evaluate the *in vitro* and *in vivo* anticancer activity of the platinum complexes. The *in vitro* growth inhibitory effect was evaluated by treating P388 cells in exponential growth phase ($10^5$cells/ml in RPMI 1640 medium supplemented with 10% foetal bovine serum and 10 μg/ml Kanamycin) with increasing concentrations of platinum complexes freshly dissolved in serum-free medium. After 1 h incubation at 37 °C, the cells were washed twice with PBS and incubated in drug-free medium for an additional 48 h; the number of viable cells was measured (trypan blue exclusion test) and growth inhibition calculated as percentage of control. $ID_{50}$ values were derived from a log-linear plot of concentration-inhibition outcomes.

The *in vivo* antileukaemic effect of platinum complexes was evaluated on the P388 leukaemia, obtained as frozen stock from the National Cancer Institute (U.S.A.), and on a cisplatin-resistant subline of P388 (P388/DDP) which was established *in vivo* in our laboratory by i.p. treatment with a single dose of cisplatin (6 mg/kg) given 2 days after the passage of $10^6$ leukaemic cells over successive generations in B6D2F1 mice (Charles River, Italy). P388 and P388/DDP cells ($10^6$/mouse) were implanted i.p. into B6D2F1 mice (6 animals/group, 8 controls). Platinum complexes were dissolved in water just before use and administered i.p. (0.1 ml per 10 g body weight) on days 1-7 at equitoxic dosages, corresponding to $LD_{0.05}$ (12). Antitumour activity was expressed as %(T/C), with T the mean survival time of treated mice and C that of untreated controls.

*Primer extension footprinting assay.* The sequence selectivity of DNA modification by platinum complexes was evaluated by the primer extension footprinting assay (13, 14). PBR 322 double stranded DNA ($1.5 \times 10^{-8}$ mol nucleotides) was reacted with platinum complexes (drug/nucleotide molar ratio = 0.02) in a total volume of 10 μl of 10 mM Tris-HCl, 1 mM EDTA, pH 8, for 1 h at 37 °C. At the end of reaction time excess drug was removed by centrifugation through Sephadex G-50 columns. After alkaline denaturation the DNA was primed with 16-mer pst(+) primer (New England Biolabs) and the synthesis performed by Sequenase 2 enzyme (United States Biochemicals) in the presence of [a-$^{32}$P]dATP (370 KBq, 111 TBq/mmol) (Amersham) and unlabeled dNTPs following the manufacturer protocol. The products of synthesis were electrophoresed (6% polyacrylamide/7 M urea gel) in parallel to a sequence ladder performed on unreacted DNA. The autoradiography was performed overnight with Kodak Ektamat G film.

*DNA interstrand cross-links.* The kinetics of interstrand cross-link formation of platinum complexes was evaluated *in vitro* by gel electrophoresis under denaturing conditions, as described by Lemaire *et al* (15). The 3000-bp pGEM-7Zf(+) DNA (Promega) linearized by Eco RI endonuclease (New England Biolabs) was mixed with platinum complexes at $(D/N)_f = 0.001$ and then incubated in 10 mM $NaClO_4$ at 37 °C. At different time intervals, the cross-linking reaction was stopped by adjusting the NaOH concentration to 10 mM and cooling the samples at - 20 °C. The samples were then analyzed on a denaturing 1% agarose gel where DNA fragments containing interstrand cross-links migrated slower than fragments without interstrand cross-links. The percentage of interstrand cross-linking was calculated from a densitometric scan of resulting bands.

### RESULTS AND DISCUSSION

The structure of acyclovir ($L^1$) is strikingly similar to that of guanosine but the rigid ribofuranosyl ring has been substituted by the more flexible 2-hydroxyethoxymethyl acyclic chain $C(1')H_2OC(2')H_2C(3')H_2OH$. Moreover two derivatives acetylated at the 2-hydroxyethoxy position ($L^2$) and at both 2-hydroxyethoxy and 2-amino positions ($L^3$) have also been used. The acetyl groups confer different solubility characteristics to the ligands and related complexes, but should not modify N(7) coordination. The synthesis and crystal structure of $[Pt(\eta^2\text{-}C_2H_4)Cl_2(L^1)]$ have been reported previously (16).

Compounds of three different types: *cis*-$[PtCl_2(NH_3)(L^1)]$, **1**, *cis*-$[PtCl_2(L)_2]$ (L = $L^2$, **2**; $L^3$, **3**) and *cis*-$[PtCl(NH_3)_2(L^1)]NO_3$ , **4**, were synthesized and characterized. The choise of these classes of compounds was based on reports of antitumour activity of compounds having similar structures (8, 17, 18).

Rochon and Kong have provided a very convenient method for the preparation of mixed amine complexes of formula *cis*-$[PtI_2(L)(L')]$. It is suggested, when synthesizing compounds of the type *cis*-$[PtI_2(NH_3)(L')]$, to start with the amine L since the $NH_3$ dimer is more difficult to obtain and the yield is poorer. However, in the case of acyclovir ($L^1$) the obtainment of the dimeric, iodo-bridged, species is considerably more difficult than with ammine. Compound **1** showed extremely low solubility even in solvents such as DMSO and DMF thus preventing the possibility of biological studies.

The synthesis of *cis*-$[PtCl_2(L)_2]$ complexes is straightforward starting from $K_2[PtCl_4]$ and free ligand in molar ratio 1:2. The yield was quantitative and gave a single pure product only in the case of $L^2$ (**2**) and $L^3$ (**3**).

*Cis*-$[PtCl(NH_3)_2(L^1)]NO_3$ (**4**) was prepared in good yield by the method of Hollis *et al.* (11).

For complexes **2-4** the NMR spectra revealed a downfield shift of the H(8) signal and, in non protic solvents, the presence of a signal attributable to N(1)H. These results indicate that in all complexes N(7) coordination occurs. Compound **1** had a very poor solubility also in solvents such as DMSO and DMF so that it could not be characterized by NMR spectroscopy. However it is assumed that the acyclovir ligand keeps the N(7) coordination found in the dimer precursor $[Pt(L^1)]_2$ from which it was obtained by bridge splitting with ammonia. Moreover IR spectra indicate the presence of chlorine ligands in cis position.

Only compound **4** had solubility in water suitable for biological investigation and therefore its antiviral and anticancer properties were investigated in comparison to those of the parent drugs.

The anti-herpes simplex-1 activity of equimolar concentrations of platinum-acyclovir and acyclovir complexes is reported in table I as mean number of cytolysis plaques and % plaque reduction on VERO cells.

Table I. Anti Herpes Simplex-1 Virus activity of acyclovir and platinum-acyclovir complex (4).

| Complex | Concentration (μM) | Plaque Number | % Plaque reduction |
|---|---|---|---|
| Vehicle | - | 256.3 | - |
| Acyclovir | 10 | 4.0 | 98.4 |
| | 1.0 | 140.7 | 45.1 |
| Pt-acyclovir (4) | 10 | 119.7 | 53.3 |
| | 1.0 | 200.3 | 21.8 |
| | 0.1 | 263.0 | 0 |

The platinum-acyclovir complex mantains the antiviral activity of the parent drug acyclovir, though showing a minor efficacy on a molar basis ($ID_{50}$ = 7.85 and 1.02 μM for platinum-acyclovir and acyclovir, respectively). This behaviour might be attributable to a minor affinity of the viral thymidine kinase and/or of the host cell kinases for the platinum-acyclovir complex as compared to acyclovir.

The effects of platinum-acyclovir complex in the murine P388 leukaemia system are reported in table II.

Table II. *In vitro* and *in vivo* antileukaemic activity (P388 system) of platinum complexes.

| | *in vitro* [a] | *in vivo* [b] | | |
|---|---|---|---|---|
| Complex | ID50 (μM) | Dose (mg/kg) | P388 | P388/DDP |
| Pt-acyclovir (4) | 108 | 50 | 209 | 140 |
| cisplatin | 2 | 0.6 | 211 | 97 |

[a] $ID_{50}$ = complex concentration inhibiting 50% cell growth.

[b] The effects on survival time of tumour-bearing mice are expressed as %T/C, *i.e.* mean survival time (x100) of treated animals versus controls.

The platinum-acyclovir complex shows an *in vitro* growth inhibitory capability, expressed as 50% growth inhibitory concentration ($ID_{50}$) markedly lower than that of cisplatin: $ID_{50}$ = 108 and 2 μM, respectively. Nevertheless, the platinum-acyclovir complex has a selective *in vivo* antitumour activity and shows the same efficacy of cisplatin in increasing the life span of animals treated with equitoxic dosages of the two componds (%T/C = 209 and 211, respectively). Moreover, compound **4** is active against a cisplatin-resistant subline of the P388 leukaemia (%T/C = 140). Similarly to cationic platinum triamine complexes synthesized by Hollis (8, 11), the platinum-acyclovir complex is less potent than cisplatin on a mole-equivalent basis. This behaviour might be related either to pharmacokinetic reasons, being platinum-acyclovir a charged compound, or to a different mechanism of action, as suggested by the absence of cross-resistance with cisplatin.

In order to address the mechanistic properties of the platinum-acyclovir complex with respect to its anticancer activity, the sequence specificity of DNA modification and the

DNA interstrand cross-linking ability were investigated and compared to those of cisplatin and $[Pt(dien)Cl]^+$, a well known antitumour-inactive platinum-triamine complex.

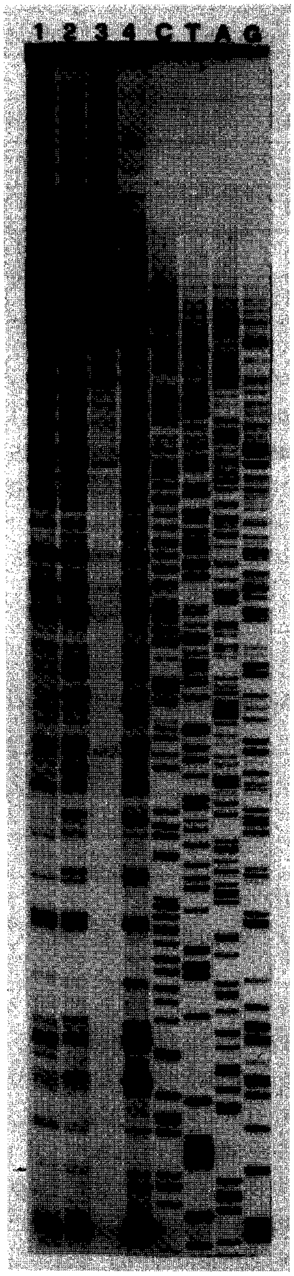

Figure 1. Primer extension footprinting assay of platinum-treated pBR322 DNA. Circular pBR322 DNA ($1.5x10^{-8}$ mol nucleotides) was reacted with platinum complexes (drug/nucleotide = 0.02) for 1 h at 37 °C. The DNA was replicated in vitro with Sequenase 2.0 enzyme and the products of synthesis were analyzed on a sequencing gel in parallel to a sequence ladder (lanes C, T, A and G) performed on unreacted DNA. Lane 1, $[Pt(dien)Cl]^+$-modified DNA; lane 2, platinum-acyclovir-modified DNA; lane 3, no reagent; lane 4, cisplatin-modified DNA.

In the primer extension footprinting assay platinum adducts on DNA template can interfere with the processivity of a polymerase, thus determining premature chain terminations corresponding to platinated sites. As shown in Figure 1, all tested compounds are able to block the Sequenase 2 enzyme and the main stop bands appear at guanine residues. In agreement with previous experiments performed with Klenow polymerase (19) and Sequenase (14) the sites of DNA synthesis termination on cisplatin-treated DNA template (lane 4) correspond to runs of two or more guanines. For d(pGG) sites polymerase stops at the first platinated nucleotide, while for the $d(pG)_5$ site (bottom of the gel) the most intense stop band, as evaluated by densitometric analysis, corresponds to the second guanine (3'-5' direction) of the template strand.

The platinum-acyclovir complex (lane 2) shows minor affinity for multiple (>2) guanines, as indicated by the low intensity of stop bands at the $d(pG)_5$ site. The main bands appear at d(pGG) sites; however, differently from cisplatin, the DNA polymerase stops at either of the two bases, and in some cases no band corresponds to d(pGG) sites, suggesting that the reactivity of adjacent guanines with Pt-acyclovir is influenced by neighbouring bases. Moreover platinum-acyclovir complex, unlike cisplatin, determines additional stop bands corresponding to cytosine residues in 3'GCT and 3'CGGC sites. The $[Pt(dien)Cl]^+$ complex, lane 1, does not determine stop bands corresponding to isolated guanines and shows poor affinity for the d(pG)5 site; moreover , like platinum-acyclovir, it determines stop bands at either of the two bases at selected d(pGG) dinucleotide sites. The intensity of stop bands on $[Pt(dien)Cl]^{+}$-treated DNA template is lower than that on cisplatin-treated template. This result does not depend upon a lower amount of platinum bound per nucleotide, indeed the experimental conditions employed resulted in a higher level of platination with respect to cisplatin (19). One possible explanation is that monofunctional adducts formed by $[Pt(dien)Cl]^{+}$ are inherently less effective than bifunctional adducts in inhibiting the DNA polymerase.

The nature of platinum-acyclovir adducts responsible for the polymerase block (*i.e.* mono- or bifunctional type) is currently under investigation. The general pattern of the footprinting experiment (more similar, in the number and intensity of stop lesions, to that of $[Pt(dien)Cl]^{+}$ than to that of cisplatin) and preliminary results obtained with terbium fluorescent probe suggest that monofunctional adducts are predominant, even though some bifunctional adducts cannot be excluded.

The kinetics of interstrand cross-link formation by platinum complexes reacting with a natural DNA fragment is shown in Figure 2.

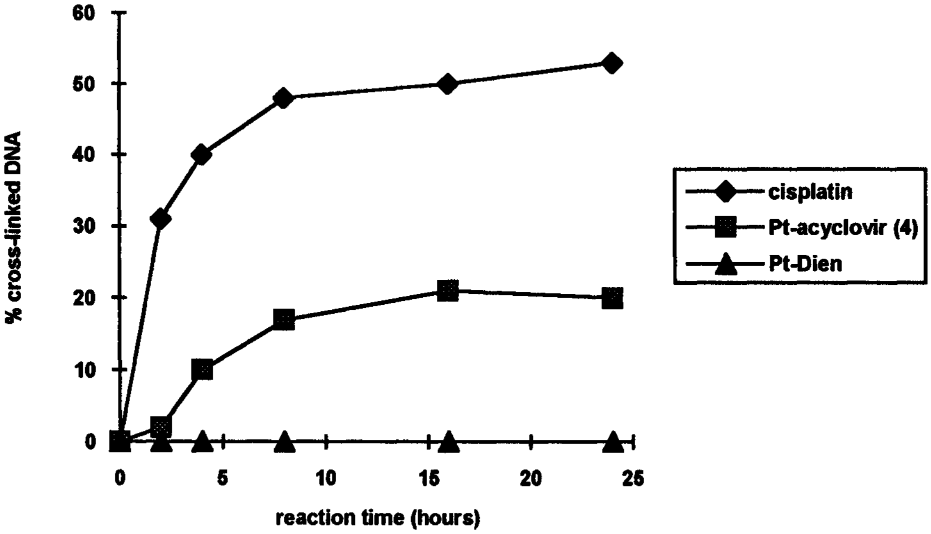

Figure 2. Kinetics of the interstrand cross-linking in the Eco RI-linearized pGEM DNA by platinum complexes (drug/nucleotide = 0.001). The samples were analyzed on a denaturing 1% agarose gel and the percentage of cross-linked DNA was calculated from a densitometric scan of resulting bands.

Both cisplatin and platinum-acyclovir complexes were effective as cross-linking agents, while the $[Pt(dien)Cl]^{+}$ complex was completely inactive. In the case of Pt-acyclovir, the rate of interstrand cross-link formation was slower than that of cisplatin, and also the extent of reaction was lower. Assuming one ICL per DNA molecule, it can be deduced that the ICLs formed by cisplatin after 24 h reaction time represented about 12% of total platinum bound, whereas the ICLs formed by platinum-acyclovir represented only 3.7%. The interstrand cross-linking capability of platinum-acyclovir complex can be explained, as reported by Leng *et al.* for the *cis*-$[Pt(NH_3)_2$(N7-N-methyl-2-diazapyrenium)$Cl]^{2+}$ complex (20), on the basis of a labilization of the aromatic base within the double-stranded DNA resulting in the formation of bifunctional adducts.

### CONCLUSION

In this work we report the synthesis of platinum(II)-acyclovir complexes and show that $[PtCl(NH_3)_2(acyclovir)]NO_3$ is endowed with both antiviral and anticancer activities. On a mole-equivalent basis the platinum-acyclovir complex is less active than the parent drugs, but it shows the same activity of cisplatin when administered at equitoxic dosages to P388 leukaemia-bearing mice. Moreover the platinum-acyclovir complex is active against the cisplatin-resistant P388 subline and is characterized by DNA interaction properties different from those of cisplatin, thus suggesting that a different mechanism of action could be operative.

**ACKNOWLEDGEMENTS**: This work was supported by the contributions of CNR, ACRO Project, MURST (40%) and EC (contract C11-CT92-0016 and COST Chemistry Project D1/02/92).

### REFERENCES

1) G.B. Elion, *Angew. Chem.*, 1989, **101**, 893.
2) E. Dubler et al., *Inorg. Chem.*, 1988, **27**, 3131.
3) E. Dubler, G. Hanggi, H. Schmalle, *Inorg. Chem.*, 1990, **29**, 2518.
4) E. Dubler, G. Hanggi, H. Schmalle, *Inorg. Chem.* 1992, **31**, 3728.
5) G. Hanggi, H. Schmalle, E. Dubler, *Inorg. Chem.*, 1993, **32**, 6095.
6) H. Mitsuya and S. Broder, *Nature*, 1987, **325**, 777.
7) S.E. Sherman and S.J. Lippard, *Chem. Rev.*, 1987, **87**, 1153.
8) L.S. Hollis, W.I. Sundquist, J.N. Burstyn, W.J. Heiger-Bernays, S.F. Bellon, K.J. Ahmed, A.R. Amundsen, E.W. Stern and S.J. Lippard, *Cancer Res.*, 1991, **51**, 1866.
9) H. Matsumoto, C. Kaneko, K. Yamada, T. Takeuchi, T. Mori and Y. Mizuno, *Chem. Pharm. Bull.*, 1968, **36**, 1153.
10) F.D. Rochon and P.C. Kong, *Can. J.Chem.*,1986, **64**, 1894.
11) L.S. Hollis, A.R. Amundsen and E.W. Sten, *J. Med. Chem.*, 1989, **32**, 128.
12) J. Litchfield and F.A. Wilcoxon, *J. Pharmacol. Exp. Ther.*, 1949, **96**, 99.
13) M. Coluccia, G. Sava, F. Loseto, A. Nassi, A. Boccarelli, D. Giordano, E. Alessio, G. Mestroni, *Eur. J. Cancer*, 1993, **29**, 1873.
14) M. Coluccia, A. Nassi, F. Loseto, A. Boccarelli, M.A. Mariggiò, D. Giordano, F.P. Intini, P. Caputo and G. Natile, *J. Med. Chem.*, 1993, **36**, 510.
15) M.A. Lemaire, A. Schwartz, A.R. Rahmouni and M. Leng, *Proc. Natl. Acad. Sci. USA*, 1991, **88**, 1982.
16) L. Cavallo, R. Cini, J. Kobe, L.G. Marzilli and G. Natile, *J. Chem. Soc. Dalton Trans.*, 1991, 1867.
17) F. K. Leh and W. Wolf, *J. Pharm. Sci.*, 1976, **65**, 315.
18) N. Farrell, O.M. Kiley, M. Schmidt and M.P. Hacker, *Inorg. Chem.*, 1990, **29**, 397.
19) A.L. Pinto and S.J. Lippard, *Proc. Natl. Acad. Sci. USA*, 1985, **82**, 4616.
20) M.F. Anin, F. Gaucheron and M. Leng, *Nucleic Acids Res.*, **1992**, 20, 4825.

**Received: June 29, 1995 - Accepted: July 27, 1997 - Accepted in revised camera-ready format: August 24, 1995**

